# Phenotypic and genomic characterization of NDM-producing *Escherichia coli* colonizing the pediatric gut in Shenzhen, China

**DOI:** 10.1128/spectrum.03329-25

**Published:** 2026-02-13

**Authors:** Yunxing He, Hongmei Yang, Cuimei Liao, Xiaoying Ou, Kaiyue Yang, Tongyan Ding, Xiaojie Zhou, Xiuju Liu, Shuyan Liu, Zhenwen Zhou, Tingjin Chen

**Affiliations:** 1 Guangdong Provincial Key Laboratory of Digestive Cancer Research, Digestive Diseases Center, Scientific Research Center, The Seventh Affiliated Hospital Sun Yat-sen University 543160 , Shenzhen, Guangdong, China; 2 Clinical Laboratory, Affiliated Shenzhen Women and Children’s Hospital (Longgang), Shantou University Medical College (Longgang District Maternity & Child Healthcare Hospital of Shenzhen City) 667494 , Shenzhen, Guangdong, China; 3 Key Laboratory of Bacterial Resistance and Prevention; Medical Research Institute of Maternal and Child, Affiliated Shenzhen Women and Children’s Hospital (Longgang), Shantou University Medical College (Longgang District Maternity & Child Healthcare Hospital of Shenzhen City) 667494 , Shenzhen, Guangdong, China; 4 Sun Yat-sen University School of Medicine, Shenzhen, Guangdong, China; 5 Clinical Laboratory, Guangzhou Women and Children's Medical Center, Guangzhou Medical University 159390, Guangzhou, Guangdong, China; Reichman University, Herzeliya, Israel

**Keywords:** carbapenem-resistant Enterobacterales, New Delhi metallo-β-lactamases, *Escherichia coli*, whole-genome sequencing, biofilm

## Abstract

Carbapenem-resistant Enterobacterales, driven by New Delhi metallo-β-lactamases (NDMs), severely restrict treatment options and are increasingly detected outside hospitals. Yet, integrated genomic–phenotypic data on NDM-positive *Escherichia coli* colonizing the pediatric gut in China remain scarce. Antimicrobial susceptibility and *bla*NDM conjugation were tested. Whole-genome sequencing (WGS) profiled the resistome, virulome, and plasmid replicon types; multilocus sequence typing (MLST), core-gene MLST, and *fimH* alleles were analyzed. We assessed biofilm-forming capacity, screened gene–biofilm associations using Benjamini–Hochberg false discovery rate, and measured cytotoxicity and siderophore activity. Twenty-five NDM-positive *E. coli* were isolated from 1,189 children stool specimens. ST48 (ST10 complex) was the most frequent sequence type (6/25, 24%); *fimH*41 was the most common *fimH* allele (4/25, 16%). All isolates were resistant to AMC, TZP, CXM, FOX, CAZ, CRO, CSL, ETP, and IMP, while none was resistant to tigecycline. All isolates transferred *bla*NDM by conjugation (frequency 1.0 × 10⁻⁴ to 9.0 × 10⁻⁴). WGS identified *bla*NDM-5 as predominant (24/25, 96%), with one *bla*NDM-1 (1/25, 4%); all isolates harbored multiple virulence and resistance genes. IncFIB (19/25, 76%) was the most common plasmid replicon. All isolates formed biofilms, including three strong producers; an exploratory screen suggested a positive association between *espL4* carriage and biofilm formation ability. All isolates increased LDH release from NCM460 cells versus the ATCC 25922 control (low cytotoxicity), without significant differences among isolates; all isolates exhibited siderophore activity as determined by Chrome Azurol S assays. NDM-positive *E. coli* colonizing children in this study were dominated by *bla*NDM-5, frequently carried IncFIB plasmids, and readily transferred *bla*NDM. All isolates showed biofilm, siderophore activity, and epithelial cytotoxicity, with espL4 positively associated with biofilm.

**IMPORTANCE:**

This study provides an integrated genomic and phenotypic characterization of pediatric gut-derived New Delhi metallo-β-lactamase (NDM)-positive *Escherichia coli* isolates. These strains were dominated by *bla*NDM-5, carried extensive resistomes, and maintained a conserved set of virulence determinants that support colonization and persistence. The frequent presence of poly-replicon plasmids, particularly IncF-type backbones, underscores their high potential for resistance dissemination. Functionally, all isolates exhibited biofilm formation, siderophore activity, and epithelial cytotoxicity, with a hypothesis-generating signal linking *espL4* to enhanced biofilm production. Together, these findings demonstrate that children can harbor high-risk, multidrug-resistant NDM-producing *E. coli* in their gut and emphasize the need for continued genomic surveillance and mechanistic studies to guide infection control and therapeutic strategies.

## INTRODUCTION

*Escherichia coli* is not only one of the most common commensal bacteria in the human intestine but also an important opportunistic pathogen responsible for a wide range of infections, including those of the urinary tract, lungs, abdominal cavity, and bloodstream (1–4). With the widespread use of carbapenems, carbapenem-resistant Enterobacterales (CRE) have rapidly emerged worldwide and are now a major concern in both clinical practice and public health. In Shenzhen, China, CRE were detected in 3.6% (90/2,474) of non-duplicate pediatric stool specimens. *E. coli* (74.5%) and *Klebsiella pneumoniae* (19.0%) were the major species, and New Delhi metallo-β-lactamase (NDM) was identified as the most common carbapenemase (5). Among these, NDM can hydrolyze nearly all β-lactams and is not inhibited by commonly used β-lactamase inhibitors. To date, 90 NDM variants have been identified (https://www.ncbi.nlm.nih.gov/pathogens/refgene/#NDM), with several variants exhibiting enhanced carbapenemase activity (6). In China, NDM-1 and NDM-5 are the predominant NDM variants in *E. coli* (7–9). Strains harboring NDM frequently display multidrug-resistant or even pan-resistant phenotypes, which severely limit therapeutic options.

Transmission of the *bla*NDM gene is largely driven by mobile genetic elements and diverse plasmid backbones, frequently co-carrying other resistance determinants such as extended-spectrum β-lactamases and 16S rRNA methyltransferases (10–14). Children may represent a vulnerable reservoir for such organisms: their gut colonization dynamics are shaped by environmental exposures, antimicrobial use, diet, and congregate settings, creating conditions that favor acquisition and onward spread of resistant strains (15, 16). However, relative to isolates from clinical infections, integrated data sets that characterize the genomic and phenotypic features of NDM-positive *E. coli* colonizing the pediatric gut in China remain scarce. Understanding these is essential for risk assessment and for designing effective interventions.

Beyond antimicrobial resistance (AMR), the pathogenic potential and colonization fitness of the strains are shaped by multiple virulence/adaptation phenotypes, including biofilm-forming capacity, high-affinity iron acquisition systems, and direct cytotoxic effects on epithelial cells (17, 18). At present, studies that determine whether NDM-positive *E. coli* colonizing the pediatric gut co-carry and express colonization/virulence phenotypes—and how these traits correspond to genomic features such as sequence type (ST) and plasmid backgrounds—are scarce. Whole-genome sequencing (WGS) provides a high-resolution framework for source tracing and risk assessment in resistant bacteria, enabling delineation of the genetic contexts of resistance genes, assignment of multilocus sequence typing (MLST), and core-gene MLST (cgMLST); when integrated with phenotypic readouts, WGS supports genotype–phenotype association analyses.

Against this background, we isolated NDM-positive *E. coli* from children’s stool samples and applied WGS to characterize their resistome, virulome, plasmid replicon types, and phylogenetic relationships. In parallel, we evaluated key *in vitro* phenotypes: cytotoxicity toward the human colonic epithelial cell line NCM460 quantified by extracellular lactate dehydrogenase (LDH) release, biofilm-forming capacity using the tissue culture plate (TCP) assay, and siderophore-mediated iron chelation using the Chrome Azurol S (CAS) methods. We also performed antimicrobial susceptibility testing. Collectively, these analyses provide an evidence base to inform infection prevention and therapeutic decision-making for pediatric populations and to support community-level surveillance and risk assessment of NDM-positive *E. coli*.

## MATERIALS AND METHODS

### Bacterial isolates

In 2024, a total of 1,189 non-duplicate fecal specimens from children were screened for carbapenem-resistant *E. coli* (CREC) at Longgang District Maternity & Child Healthcare Hospital of Shenzhen City, China. The samples were streaked individually onto MacConkey agar plates containing 1.5 μg/mL imipenem and incubated for 48 h at 37°C with 5% CO_2_. These strains were then verified using primers reported in Poirel’s study (19) (NDM-F: 5′-GGTTTGGCGATCTGGTTTTC-3′; NDM-R: 5′-CGGAATGGCTCATCACGATC-3′), followed by screening to identify NDM-positive strains. Finally, this subset of NDM-positive strains was incorporated into the present study.

### Antimicrobial sensitivity testing

We tested all isolates for sensitivity to amoxicillin–clavulanic acid (AMC), piperacillin/tazobactam (TZP), cefuroxime (CXM), cefoxitin (FOX), ceftazidime (CAZ), ceftriaxone (CRO), cefoperazone–sulbactam (CSL), cefepime (FEP), ertapenem (ETP), imipenem (IPM), amikacin (AMK), levofloxacin (LVX), tigecycline (TGC), and trimethoprim/sulfamethoxazole. Antimicrobial sensitivity testing was performed by broth microdilution on a VITEK 2 Compact using VITEK 2 AST cards (bioMérieux, France). Results were interpreted based on the Clinical and Laboratory Standards Institute (CLSI) Performance Standards for Antimicrobial Susceptibility Testing, 34th ed (20). CLSI-recommended ATCC 25922 and ATCC 700603 strains were used as controls.

### WGS and bioinformatics analysis

The genomic DNA was extracted with a commercial kit and sequenced using PacBio Sequel and Illumina NovaSeq 6000. Reads were filtered and assembled using SPAdes (https://github.com/ablab/spades; v3.15.5) (21), fastp (https://github.com/OpenGene/fastp) (22), and gtdbtk (https://gtdb.ecogenomic.org/) (23) (v2.3.2). Plasmid replicon types were identified using PlasmidFinder 2.0 with an identity threshold of 95% and a minimum coverage length of 60% (24). Acquired AMR genes were identified with Comprehensive Antibiotic Resistance Database (25). Virulence-associated genes were identified by screening draft assemblies against the Virulence Factor Database with thresholds of ≥95% nucleotide identity and ≥60% coverage (26).

### Cell culture and *E. coli* infection

Cytotoxicity was evaluated by measuring the release of LDH, a stable cytosolic enzyme released upon plasma membrane damage marker. NCM460 human colonic epithelial cells were cultured in Dulbecco’s modified Eagle’s medium containing 10% fetal bovine serum in a 5% CO_2_ incubator. Cells were seeded in 24-well plates at a density of 1 × 10⁵ cells per well and incubated for 24 h. The cell-culture medium was then supplemented with 2 × 10⁷ CFU of *E. coli* and incubated for 24 h. After centrifugation at 3,000 rpm and 4°C for 5 min, the supernatant was analyzed for the LDH Cytotoxicity Assay kit (Solarbio, BC0685), according to the manufacturer’s instructions. *E. coli* ATCC 25922 served as the reference strain (low cytotoxicity), whereas the blank control group (culture medium without bacteria) was used for blank correction.

### Conjugation assay

The conjugation experiments were performed using rifampicin-resistant EC600 as the recipient strain and NDM-producing *E. coli* as the donor strain. The donor and recipient strains were inoculated onto Luria–Bertani (LB) plates for 18–24 h. Five to nine fresh single colonies were picked and inoculated into 5 mL of LB medium and incubated in a shaker at 37°C for 3.5–4.0 h. Equal volumes of donor bacteria (100 µL) and recipient bacteria (100 µL) were mixed with 1 mL of fresh LB medium and co-incubated for 18–24 h at 37°C. Transconjugants were selected on LB plates supplemented with rifampicin (100 µg/mL) and imipenem (4 µg/mL). The presence of the *bla*NDM gene in transconjugants was confirmed by PCR as described above. The conjugation transfer frequency was calculated as the number of transconjugant colonies divided by the number of recipient colonies.

### Quantitative siderophore production assay

To assess the ability of bacterial supernatants to chelate iron, the researchers utilized the CAS assay, following established protocols (27). For CAS solid-state detection, specifically, 10 mL of stationary-phase, iron-chelated cultures was deposited on CAS plates (Beijing Coolabo Technology, China), and after 48 h of incubation at 37°C, the presence of orange halos was used to identify siderophore production. CAS liquid detection: briefly, overnight cultures in LB broth were diluted 1:100 into M9 minimal medium supplemented with 0.2% glucose and incubated at 37°C with shaking (180 rpm) for 48 h to induce siderophore expression. Cell-free supernatants were obtained by centrifugation (10,000 × *g*, 10 min) followed by filtration through 0.22 µm filters. The supernatant (100 µL) was mixed with an equal volume of CAS detection solution (Beijing Coolabo Technology) in 96-well plates. After incubation at room temperature for 30 min in the dark, absorbance was measured at 630 nm using a Multiskan GO microplate reader (Thermo Fisher Scientific). Sterile M9 medium mixed 1:1 with CAS solution served as the blank (Ar). ATCC 25922 served as negative reference control (As). Siderophore activity was expressed as percentage of color change calculated using the formula [(Ar − As) / Ar] × 100%. Each isolate was tested in three independent biological replicates.

### Detection of biofilm formation ability

The TCP method was adapted from Choi and Ko (28). Briefly, a 0.5 McFarland bacterial suspension of the biofilm-positive strains was prepared. The suspensions were diluted 1:100 with fresh LB broth, and 200 μL of this diluted culture was added to a 96-well plate (each strain in triplicate, with LB broth alone as negative control). The plates were incubated at 37°C for 18–24 h. After incubation, the liquid in the wells was aspirated out after centrifugation, and 200 μL sterile water was used to gently wash the bacteria three times and air-dried. Biofilms formed in the wells were fixed with 200 μL formaldehyde for 5 min and stained with 200 μL 1% crystal violet for 15 min, then washed twice with sterile water and air-dried. After drying, the dried crystal violet stain was dissolved in 200 μL absolute ethanol. The OD of the solution was measured at 595 nm using a microplate reader, Multiskan GO (Thermo Fisher Scientific, USA). A mean of OD values was calculated for each strain (ODs), while the cut-off OD (ODc) was calculated as three standard deviations above the mean OD of the negative control (29). The extent of biofilm formation was classified as strong (ODs > ODc), moderate (2 ODc’s < ODs ≤ 4 ODc’s), weak (ODc < ODs ≤ 2 ODc’s), and none (ODs ≤ ODc) (30). The experiment was performed in triplicate, and the final value was the average outer diameter of the attached well.

### MLST and *fimH* typing

STs of *E. coli* were determined using the Achtman seven-locus scheme (*adk*, *fumC*, *gyrB*, *icd*, *mdh*, *purA*, and *recA*) (31). To analyze the phylogenetic relationships among target strains, the MLST results were phylogenetically visualized using the GrapeTree tool, generating a clear clustering tree of strain genotypes (32). Assembled genomes were queried with the MLST scheme against the *E. coli* database in EnteroBase to assign allele numbers and STs. Whole-genome sequencing results were subjected to *fimH* typing using FimTyper (v1.0) (33).

### False discovery rate control

To account for multiple hypothesis testing in the exploratory screens, Benjamini–Hochberg (BH) false discovery rate (FDR) control was applied to the family of *P* values generated within each screen. Specifically, for the gene-level screen, we tested each binary virulence gene (present/absent) against the continuous outcome using two-sided Mann–Whitney *U* tests and then computed BH-adjusted *q* values across all genes. A significance threshold of *q* < 0.10 was pre-specified; nominal *P* values are reported alongside *q* values. For pathway-restricted (candidate) analyses, BH adjustment was repeated within the subset. For the exploratory double-positive (GeneA∧GeneB) screen, BH adjustment was likewise applied across all tested pairs, and results were interpreted as hypothesis generating. Calculations were performed in Python (SciPy/statsmodels).

### Statistics

Statistical significance was assessed using a two-sided Student’s *t*-test and Mann–Whitney *U* (non-parametric unpaired test) of the GraphPad Prism 8 software. *P* < 0.05 was considered statistically significant.

## RESULTS

### Lineages, resistance, and conjugative transfer of pediatric NDM-producing *E. coli*

**Fig 1:**
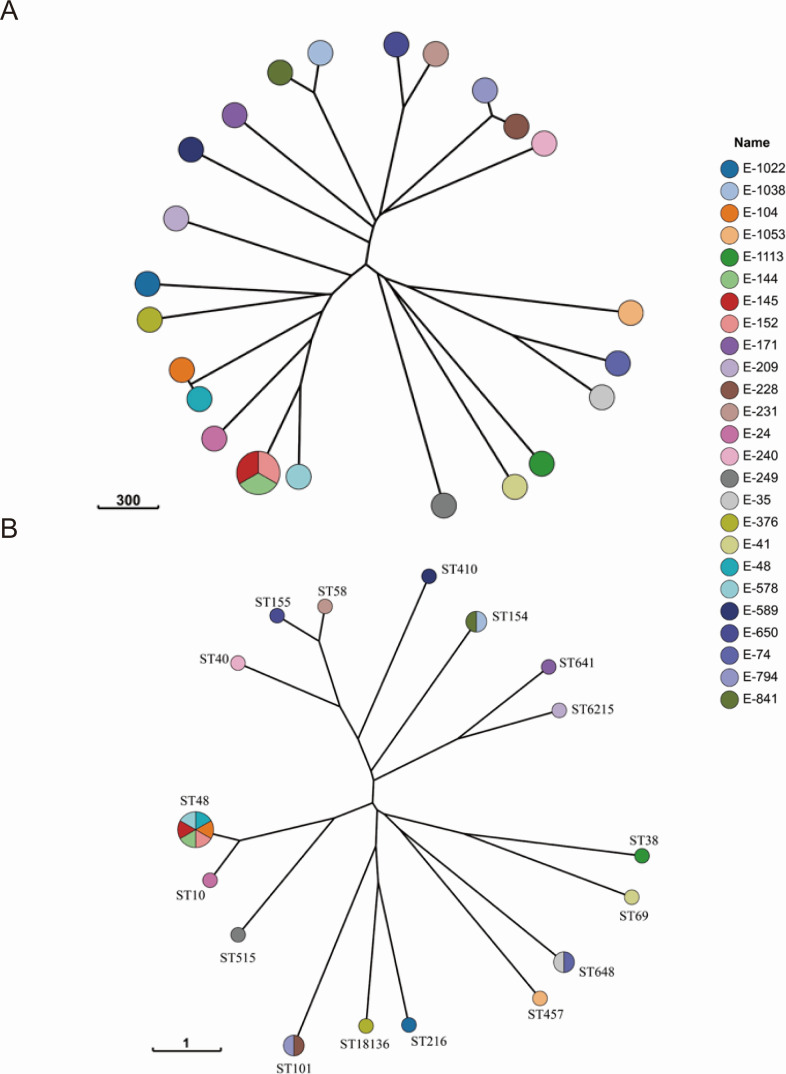
GrapeTree minimum-spanning tree of CREC isolates constructed using the neighbor-joining algorithm implemented in EnteroBase. (**A**) Tree based on core genome multilocus sequence typing. (**B**) Tree based on classical seven-gene multilocus sequence typing. Each node represents one or more isolates (displayed as pie charts when multiple isolates collapse into the same node) and is colored by isolate ID. Branch labels indicate the number of allelic differences (genetic distance).

We isolated a total of 25 NDM-positive *E. coli* from 1,189 samples (Table 1; Fig. 1). Patients ranged from neonates (11 days) to late adolescence (16.8 years), with a predominance of males. The dominant lineage was ST48 (ST10 Cplx; *n* = 6, 24.0%), followed by ST648 (ST10 Cplx; *n* = 2, 8.0%), ST101 (ST101 Cplx; *n* = 2, 8.0%), and ST155 (ST155 Cplx; *n* = 2, 8.0%) among 25 isolates. We also identified a novel ST (ST18136); allele profiles are shown in Table 2. However, only three strains of ST48 belong to the same cgMLST (Fig. 1). The most frequent alleles were *fimH*41 (*n* = 4, 16.0%), following *fimH*23, *fimH*31, and *fimH*54 (each *n* = 3, 12.0%), with additional types (e.g., *fimH*24, *fimH*86, *fimH*32, *fimH*30, *fimH*69, and *fimH*145) and one *fimH*58-like.

**TABLE 1:** Antimicrobial susceptibility profiles of 25 NDM-producing *E. coli* isolates

No.	Sex	MLST (clonal complex)^*a*^	*fimH* typing	Age (year)	MIC (μg/mL)^*b*^														Conjugation frequency^*c*^
					AMC	TZP	CXM	FOX	CAZ	CRO	CSL	FEP	ETP	IPM	AMK	LVX	TGC	SXT	
24	Female	10 (ST10 Cplx)	*fimH*23	13 years	≥32	≥128	≥64	≥64	≥64	≥64	≥64	16	≥8	8	4	1	≤0.5	≤20	2.46 × 10^−4^
35	Male	648 (ST10 Cplx)	*fimH*24	9 months and 25 days	≥32	≥128	≥64	≥64	≥64	≥64	≥64	16	≥8	≥16	4	≤0.12	≤0.5	≥320	3.59 ×10^−4^
41	Male	69 (ST69 Cplx)	*fimH*27	4 years, 8 months, and 1 day	≥32	≥128	≥64	≥64	≥64	≥64	≥64	16	2	≥16	≤2	1	≤0.5	≥320	6.45 × 10^−4^
48	Male	48 (ST10 Cplx)	*fimH*23	3 years	≥32	≥128	≥64	≥64	≥64	≥64	≥64	16	≥8	≥16	≤2	1	≤0.5	≥320	1.22 × 10^−4^
74	Male	648 (ST10 Cplx)	*fimH*58-like	1 year, 9 months, and 29 days	≥32	≥128	≥64	≥64	≥64	≥64	≥64	16	≥8	≥16	≤2	≥8	≤0.5	≥320	1.45 × 10^−4^
104	Male	48 (ST10 Cplx)	*fimH*23	13 years, 8 months, and 1 day	≥32	≥128	≥64	≥64	≥64	≥64	≥64	16	≥8	≥16	≤2	1	≤0.5	≥320	5.64 × 10^−4^
144	Male	48 (ST10 Cplx)	*fimH*41	3 months and 29 days	≥32	≥128	≥64	≥64	≥64	≥64	≥64	16	≥8	≥16	≤2	1	≤0.5	≥320	3.66 × 10^−4^
145	Male	48 (ST10 Cplx)	*fimH*41	1 year	≥32	≥128	≥64	≥64	≥64	≥64	≥64	16	≥8	≥16	≤2	1	≤0.5	≥320	4.16 × 10^−4^
152	Female	48 (ST10 Cplx)	*fimH*41	2 months and 1 day	≥32	≥128	≥64	≥64	≥64	≥64	≥64	16	≥8	≥16	≤2	1	≤0.5	≥320	8.81 × 10^−4^
171	Male	641 (ST86 Cplx)	*fimH*54	3 years, 9 months, and 29 days	≥32	≥128	≥64	≥64	≥64	≥64	≥64	16	≥8	≥16	≤2	1	≤0.5	≤20	7.69 × 10^−4^
209	Male	6,215 (−)	*fimH*34	5 years	≥32	≥128	≥64	≥64	≥64	≥64	≥64	16	≥8	≥16	≥64	≥8	≤0.5	≥320	4.34 × 10^−4^
228	Male	101 (ST101 Cplx)	*fimH*86	2 years	≥32	≥128	≥64	≥64	≥64	≥64	≥64	16	≥8	≥16	≤2	≤0.12	≤0.5	≥320	2.56 × 10^−4^
231	Male	58 (ST155 Cplx)	*fimH*32	8 months and 1 day	≥32	≥128	≥64	≥64	≥64	≥64	≥64	16	≥8	≥16	≤2	1	≤0.5	≥320	1.00 × 10^−4^
240	Female	40 (ST40 Cplx)	*fimH*31	6 years, 3 months, and 29 days	≥32	≥128	≥64	≥64	≥64	≥64	≥64	16	≥8	≥16	≤2	≤0.12	≤0.5	≤20	6.18 × 10^−4^
249	Male	515 (−)	*fimH*41	1 year	≥32	≥128	≥64	≥64	≥64	≥64	≥64	16	≥8	≥16	≤2	2	≤0.5	≥320	4.45 × 10^−4^
376	Male	18,136 (ST168 Cplx)	–	11 days	≥32	≥128	≥64	≥64	≥64	≥64	≥64	16	≥8	≥16	≤2	1	≤0.5	≤20	4.99 × 10^−4^
578	Female	48 (ST10 Cplx*fimH*)	*fimH*54	13 years, 9 months, and 29 days	≥32	≥128	≥64	≥64	≥64	≥64	≥64	4	≥8	≥16	≤2	1	4	≥320	5.12 × 10^−4^
589	Male	410 (ST23 Cplx)	*fimH*24	6 years, 2 months, and 1 day	≥32	≥128	≥64	≥64	≥64	≥64	≥64	16	≥8	≥16	4	1	≤0.5	≥320	6.74 × 10^−4^
650	Female	155 (ST155 Cplx)	*fimH*30	4 years and 6 months	≥32	≥128	≥64	≥64	≥64	≥64	≥64	8	≥8	≥16	≤2	1	≤0.5	≥320	3.29 × 10^−4^
794	Female	101 (ST101 Cplx)	*fimH*86	7 years, 8 months, and 1 day	≥32	≥128	≥64	≥64	≥64	≥64	≥64	16	≥8	≥16	≤2	≤0.12	≤0.5	≤20	3.17 × 10^−4^
841	Male	154 (−)	*fimH*31	1 year and 29 days	≥32	≥128	≥64	≥64	≥64	≥64	≥64	16	≥8	≥16	≤2	1	≤0.5	≤20	6.88 × 10^−4^
1,022	Male	216 (−)	*fimH*69	11 years, 11 months, and 1 day	≥32	≥128	≥64	≥64	≥64	≥64	≥64	16	≥8	≥16	≤2	1	≤0.5	≥320	5.35 × 10^−4^
1,038	Male	154 (−)	*fimH*31	1 year, 8 months, and 1 day	≥32	≥128	≥64	≥64	≥64	≥64	≥64	16	≥8	≥16	≤2	1	≤0.5	≥320	8.85 × 10^−4^
1,053	Male	457 (−)	*fimH*145	1 year, 11 months, and 1 day	≥32	≥128	≥64	≥64	≥64	≥64	≥64	16	≥8	≥16	4	≥8	≤0.5	≥320	7.45 × 10^−4^
1,113	Male	38 (ST38 Cplx)	*fimH*54	16 years, 9 months, and 29 days	≥32	≥128	≥64	≥64	≥64	≥64	≥64	16	≥8	≥16	≤2	1	≤0.5	≤20	8.97 × 10^−4^

^*a*^ Cplx, clonal complex; MLST, multilocus sequence typing.

^*b*^ Antibiotic abbreviations: AMC, amoxicillin–clavulanate; TZP, piperacillin–tazobactam; CXM, cefuroxime; FOX, cefoxitin; CAZ, ceftazidime; CRO, ceftriaxone; CSL, cefoperazone–sulbactam; FEP, cefepime; ETP, ertapenem; IPM, imipenem; AMK, amikacin; LVX, levofloxacin; TGC, tigecycline; SXT, trimethoprim–sulfamethoxazole. A dash (–) indicates not determined.

^*c*^ Conjugation assays targeted transfer of *bla*NDM-bearing plasmids to a laboratory *E. coli* recipient; conjugation frequency was calculated as the number of transconjugants per recipient cell.

**TABLE 2:** The allelic profiles of the novel STs found in this study are shown below

ST	*adk*	*fumC*	*gyrB*	*icd*	*mdh*	*purA*	*recA*
18,136	1,398	11	4	10	7	7	6

All isolates were resistant to AMC, TZP, CXM, FOX, CAZ, CRO, and CSL. With respect to FEP, one isolate (strain 578) exhibited an intermediate phenotype, whereas all others were resistant. All isolates were resistant to the carbapenems ETP and IPM. No resistance to TGC was observed, and only one isolate was resistant to AMK (1/25, 4.0%). Resistance to LVX was relatively low (3/25, 12.0%), whereas a high rate of resistance to SXT was detected (18/25, 72.0%). All isolates successfully transferred the *bla*NDM gene by conjugation, with transfer frequencies on the order of 10^−^⁴ per recipient (range, 1.0×10^−^⁴ to 9.0×10^−^⁴), indicating efficient plasmid-mediated dissemination potential.

### Distribution of virulence, plasmid replicons, and resistance genes

#### Resistance genes

**Fig 2:**
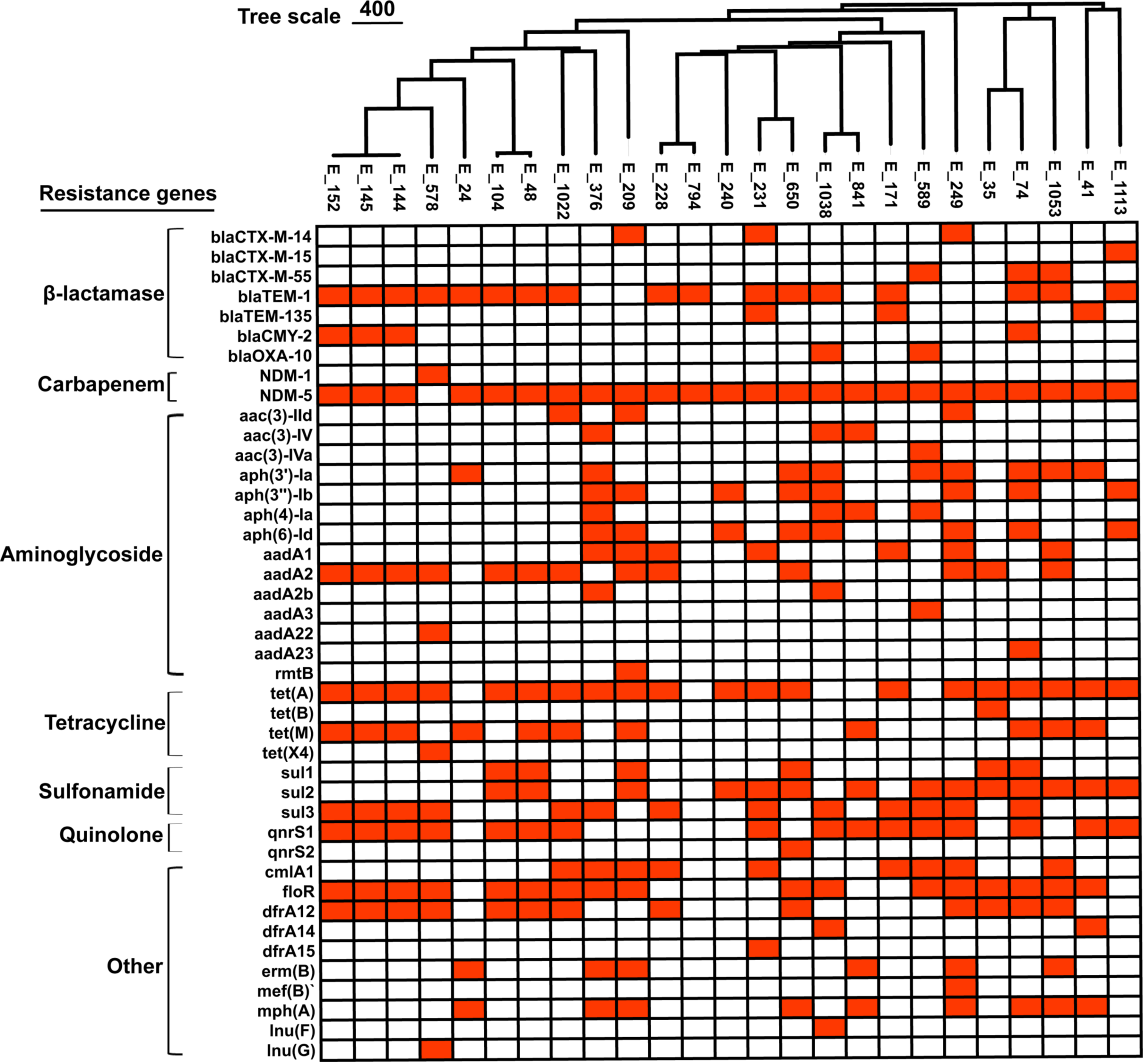
Distribution of resistance genes based on cgMLST clustering.

Among the 25 isolates, the carbapenemase gene NDM-5 was detected in 24 isolates (96%), whereas NDM-1 was identified in only 1 isolate (4%) (Fig. 2). β-Lactamases were dominated by *blaTEM-1* (17/25, 68%), with *blaCTX-M-14* and *blaCTX-M-55* each in 3/25 (12%), *blaTEM-135* in 3/25 (12%) and *blaCTX-M-15* in 1/25 (4%). Tetracycline resistance genes were frequent: *tet*(*A*) and *tet(M*) occurred in 20/25 (80%) and 11/25 (44%), respectively. The plasmid-mediated quinolone resistance gene *qnrS1* was present in 16/25 (64%). Sulfonamide genes *sul2* and *sul3* were found in 14/25 (56%) and 13/25 (52%), respectively, and the trimethoprim gene *dfrA12* was found in 13/25 (52%). Aminoglycoside determinants included *aadA2* in 13/25 (52%), *aph(3′)-Ia* in 9/25 (36%), and *aph(3″)-Ib* and *aph(6)-Id* each in 8/25 (32%). Phenicol resistance genes were also common, with *floR* and *cmlA1* identified in 17/25 (68%) and 9/25 (36%), respectively. All isolates carried multiple resistance genes; specifically, 3/25 (12%) harbored ≥16 genes; 9/25 (36%) carried 11–15 genes; 11/25 (44%) carried 6–10 genes; and 2/25 (8%) carried ≤5 genes.

#### Virulence genes

**Fig 3:**
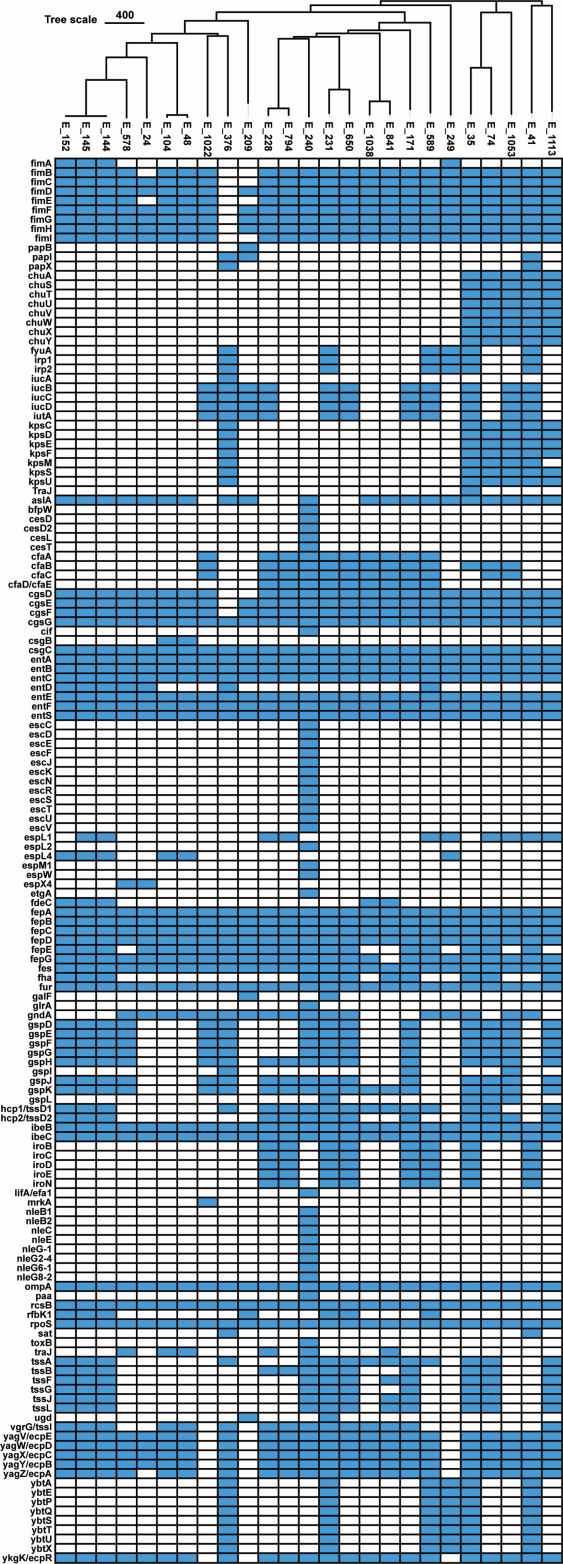
Distribution of virulence genes based on cgMLST clustering.

Among the 25 isolates, virulence genes exhibited heterogeneous distribution (Fig. 3). Nineteen genes were universally detected (100% prevalence), including *cgsG*, *csgC*, *entA*, *entB*, *entC*, *entE*, *entF*, *entS*, *fepA*, *fepB*, *fepC*, *fepD*, *fes*, *fur*, *ibeB*, *ibeC*, *ompA*, *rcsB*, and *rpoS*. In total, 38 virulence genes showed high prevalence (≥80%), encompassing fimbrial operon genes (*fimB*–*fimI*), curli-associated genes (*cgsD*–*cgsG*), and siderophore-related genes (*entA*–*entF*, *entS*, and *fepC*). Fourteen genes were detected at moderate prevalence (50%–79%), including *cfaB*, *fepE*, and *gndA*, several type II secretion system genes (*gspD*–*gspK*), and type VI secretion system components (*hcp1*/*tssD1*, *hcp2*/*tssD2*, *tssA*, and *vgrG*/*tssI*). In contrast, the majority of virulence determinants (98/150) displayed low prevalence (<50%).

#### Plasmid replicons

**Fig 4:**
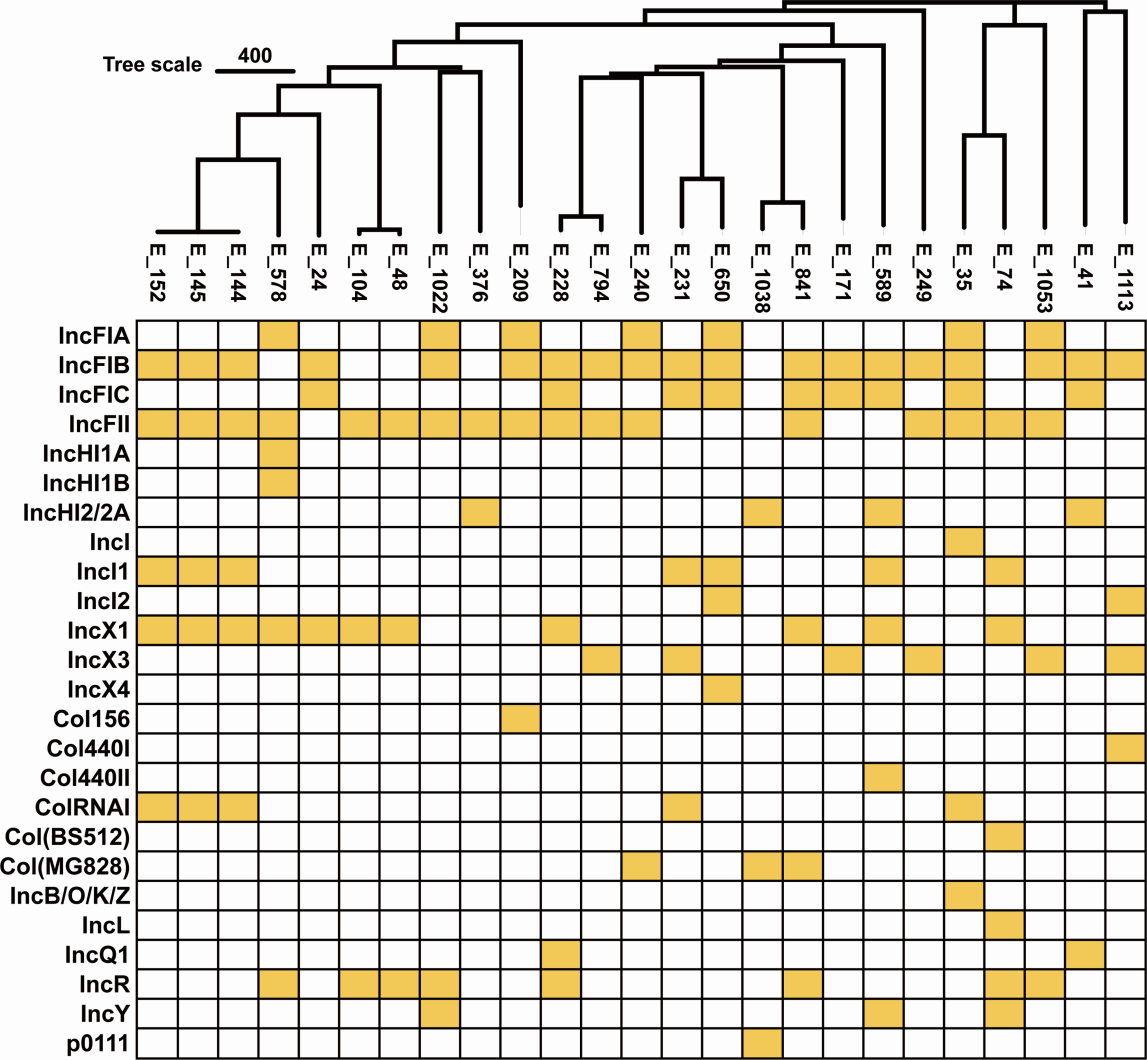
Distribution of plasmid replicons based on cgMLST clustering.

Plasmid replicon analysis showed a predominance of IncF-type backbones: IncFIB was detected in 19/25 isolates (76%) and IncFII in 17/25 isolates (68%) (Fig. 4). Other replicons included IncX1 (11/25, 44%), IncFIC (9/25, 36%), IncR (8/25, 32%), and IncI1 and IncFIA (7/25, 28% each). Less frequent types were IncX3 (6/25, 24%), ColRNAI (5/25, 20%), IncHI2 and IncHI2A (4/25, 16% each), and IncY and Col(MG828) (3/25, 12% each). Rare replicons (≤2/25, ≤8%) included IncQ1, IncI2, IncL, IncB/O/K/Z, IncX4, Col(BS512), Col440I/II, Col156, p0111, IncI, IncHI1A, and IncHI1B. Most isolates carried ≥5 replicons (14/25, 56%), with 4 replicons in 4/25 (16%) and 3 replicons in 7/25 (28%); none harbored ≤2 replicons.

### *espL4*-positive isolates exhibit higher biofilm formation

**Fig 5:**
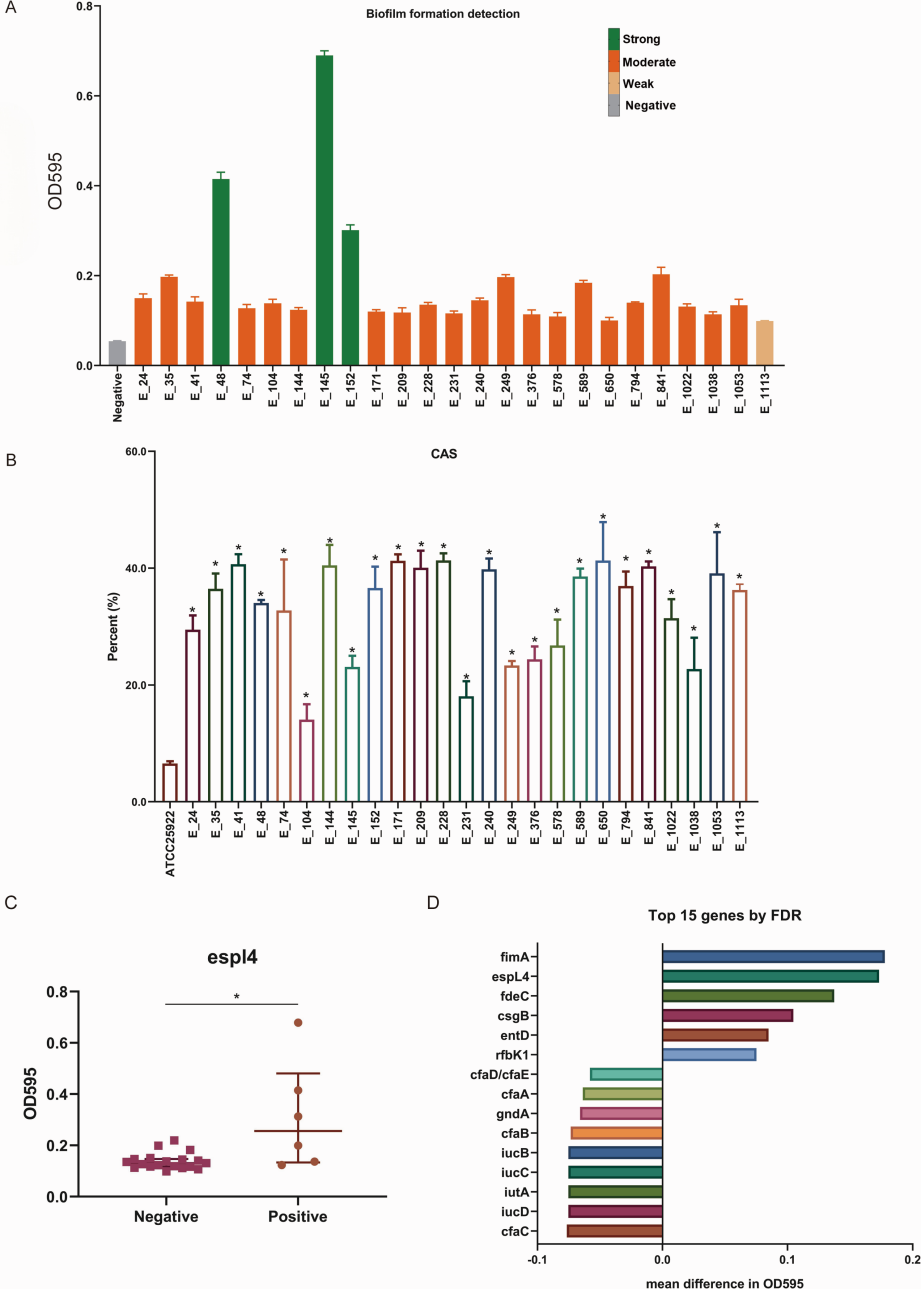
Gene-level screening and confirmation. (**A**) Biofilm formation for each isolate was quantified by TCP assay as absorbance at 595 nm (**B**) Siderophore production by 25 NDM-producing *E. coli* isolates as determined by the CAS assay. *P < 0.05. (**C**) Distribution of OD595 for *espL4*-positive (*n* = 6) and *espL4*-negative (*n* = 19) isolates. Points represent individual isolates; horizontal lines and whiskers denote the median and interquartile range. (**D**) Top 15 virulence genes ranked by Benjamini–Hochberg FDR from two-sided Mann–Whitney *U* tests comparing the continuous OD595 measurement between gene-positive and gene-negative isolates (*n* = 25). Bars show the mean difference (positive − negative).

Figure 5A shows biofilm formation in 25 isolates measured by the TCP microtiter assay at OD595. All isolates produced biofilm. According to TCP criteria, three isolates (152, 145, and 48; 3/25, 12.0%) were classified as strong producers, the remainder (21/25, 84.0%) as moderate producers, and one isolate (1,113; 1/25, 4.0%) as a weak producer. We then performed a discovery screen testing each detected virulence gene (present/absent) against the OD595 (Fig. 5D). Bars display the mean difference in OD595 between gene-positive and gene-negative isolates (positive−negative), ranked by Benjamini–Hochberg FDR. No gene remained significant after FDR control (*q* < 0.10). Nevertheless, *espL4* showed the largest positive effect size and the lowest *q*, with fimA exhibiting a similar positive trend, whereas several cfa loci tended to be lower. Given this leading signal, *espL4* was pre-specified for confirmatory testing (Fig. 3C). OD595 values were higher in espL4-positive isolates (*n* = 6) than in espL4-negative isolates (*n* = 19): median (IQR) 0.257 (0.153–0.389) versus 0.131 (0.118–0.146); two-sided Mann–Whitney *P* = 0.0172. The relationship between the remaining 14 genes and biofilm formation ability is shown in Fig. S1.

### Virulence was observed across all isolates

**Fig 6:**
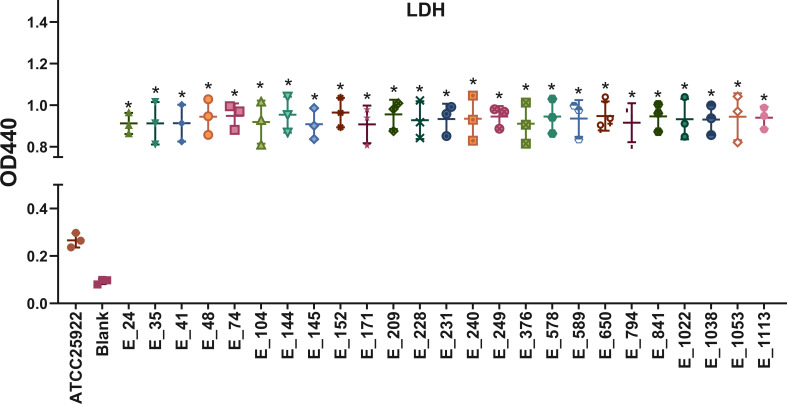
The LDH activity in NCM460 infected by different strains. An unpaired two-sided Student’s *t*-test was performed for LDH production (mean ± standard). All isolates showed significantly higher cytotoxicity than the reference strain *E. coli* ATCC 25922 (low cytotoxicity, unpaired two-sided Student’s *t*-test, *P < 0.05).

All 25 NDM-producing *E. coli* isolates exhibited strong siderophore production in the quantitative liquid CAS assay, with color change percentages ranging from 14.1% to 41.4%. All isolates produced significantly higher siderophore activity than ATCC 25922 (6.6%, *P* < 0.05; Fig. 5B; Fig. S2). Figure 6 shows LDH release in the supernatant of NCM460 cells following exposure to each isolate. Relative to the ATCC 25922 reference strain, all isolates induced higher LDH levels (*P* < 0.05), whereas no statistically significant differences were detected among isolates.

## DISCUSSION

Antimicrobial resistance is a global public health emergency (34). Among the few options for multidrug-resistant gram-negative infections, carbapenems remain pivotal last-line β-lactams (35). A global analysis of CREC from human sources (2005–2023) showed that isolates were reported mainly from the United States (17.49%), China (14.88%), and the United Kingdom (14.73%) (36). From 2019 to 2023, the adjusted annual incidence of clinical cultures positive for CRE in the United States increased by 69% (37). The study in the Shenzhen region of China found a CREC carriage rate of 2.7% in stool samples from hospitalized children (5). Similarly, a 2023 nationwide Chinese study on pediatric diarrhea stool samples reported an isolation rate of 1.63% (38). In contrast, a Japanese study involving 300 stool samples from infants aged 4–5 months detected no CREC isolates (39). NDM was the predominant carbapenemase worldwide; among NDM variants, NDM-1 and NDM-5 were dominant—76.24% and 15.33%, respectively—together accounting for >90% of NDM-positive isolates. In China, NDM-5 and NDM-1 were the leading circulating carbapenemase in CREC (7, 8, 36, 40). In China, NDM-5 has been reported predominantly in ST167, whereas NDM-1 is mainly associated with ST131 (36). However, in our cohort, NDM-5 was most frequent in ST48 (5/24), and the single NDM-1 isolate also belonged to ST48. Furthermore, cgMLST clustering analysis revealed that strains 144, 145, and 152 share identical cgMLST features and carry the same resistance genes and plasmid replicons. However, virulence gene identification (based on ≥95% nucleotide identity and ≥60% coverage) showed that strain 152 lacks the espL1 virulence gene. Given the limited sample size (single-center pediatric carriage cohort), these findings may not fully represent the dominant sequence types in children from Shenzhen Longgang; larger, ongoing surveillance is warranted. Our results showed that nearly all isolates were resistant to β-lactam antibiotics, with only one strain exhibiting intermediate resistance to FEP. This finding is consistent with previous reports (41–43). In our earlier study on the epidemiology of carbapenem-resistant Enterobacteriaceae among pediatric inpatients in South China (44), no TGC-resistant strains were detected. Notably, intestinal carriage of CREC in children in our study was largely susceptible to levofloxacin, with only 12% resistant. This rate is markedly lower than that reported in our previous inpatient study (67.5%) and also lower than the findings of Xu et al. (45). The discrepancy may be explained by differences in age distribution (12.0–62.5 years in the earlier cohort versus 11 days–16.8 years in the present study) and by the lower exposure of younger children to fluoroquinolones, which are generally restricted in pediatric practice. These results suggest that age-related antibiotic usage patterns may influence resistance profiles, highlighting the need for continued surveillance across diverse pediatric populations. One limitation is the absence of susceptibility testing for newer agents such as aztreonam/avibactam and cefiderocol, which were not part of the routine diagnostic panel at the time of isolate collection. Future prospective studies should include these compounds to better guide therapeutic options.

The previous studies showed that *bla*TEM and *bla*CTX-M families frequently co-occur with *bla*NDM in clinical and colonizing *E. coli*, thereby compounding multidrug resistance (6). Our genomic analysis revealed a broad resistome among pediatric NDM-positive *E. coli*. β-Lactamases were dominated by *bla*TEM-1 (68%), with additional ESBLs such as *bla*CTX-M-14, *bla*CTX-M-55, and *bla*CTX-M-15, as well as *bla*TEM-135. Beyond β-lactams, this study determinant genes were widely distributed against tetracycline, quinolone, sulfonamide, trimethoprim, multiple aminoglycoside-modifying enzymes, and phenicol. The accumulation of such determinants underscores the role of mobile genetic elements in shaping multidrug resistance in NDM-positive strains, highlighting the formidable therapeutic challenges these strains present. All 25 isolates harbored multiple acquired resistance genes, with 22 (88%) carrying ≥10 determinants besides *bla*NDM. While these strains represent intestinal colonization rather than active clinical infection, their broad resistance repertoire highlights the potential difficulty in treating possible future invasive infections caused by such clones.

The virulence gene profile was similarly complex. We found 19 core virulence genes universally present across all isolates, mainly involved in adhesion (curli fimbriae, fim operon) and iron acquisition (enterobactin and ferric-enterobactin transport systems). Such genes are regarded as essential for intestinal colonization and survival in iron-limited environments (46–48). In clinical *E. coli*, the isolation rate of fim is as high as 92.2%, among which the proportion in *E. coli* isolated from urine is as high as 100% (49). However, our research shows that all strains except strain 376 carry one or more fim genes. Meanwhile, our results also showed that all CREC isolates were positive in the CAS assay, and the iron chelation rates of all strains were significantly higher than that of ATCC 25922. A large set of additional genes also showed high prevalence (≥80%), including curli operon components and siderophore biosynthesis clusters, suggesting that carriage strains are well equipped for mucosal persistence. Moderate-prevalence genes (50%–79%), such as cfaB and type II/VI secretion system components, indicate heterogeneity in virulence potential across lineages. Importantly, the majority of virulence determinants were sporadic (<50%), reflecting a mosaic distribution of accessory pathogenicity factors. Taken together, our data highlight that pediatric NDM-positive *E. coli* combine extensive resistance repertoires with conserved virulence backbones, ensuring both multidrug resistance and colonization fitness. This dual profile highlights the potential public health threat posed by these strains and emphasizes the importance of integrated genomic surveillance of both resistome and virulome in pediatric populations. Moreover, LDH release serves as a marker of cell damage (50), and in our study, all isolates induced LDH secretion from NCM460 cells, with levels three- to fourfold higher than the reference strain ATCC 25922, supporting their potential to cause epithelial injury in addition to harboring diverse virulence determinants.

Plasmids have become the principal vehicles of antimicrobial resistance. Through processes of microevolution and fusion, modern plasmids have developed into highly recombinogenic, multireplicon, self-transmissible elements that represent a major driver of resistance dissemination and a significant threat to human health (51, 52). In our cohort, WGS showed that isolates typically carried multiple plasmid backbones (mean 4.7 replicons per genome). Replicon profiling revealed a predominance of IncF-type scaffolds—IncFIB (19/25, 76%) and IncFII (17/25, 68%). These findings are congruent with contemporary genomic epidemiology in which IncF backbones frequently dominate *E. coli* populations and act as versatile AMR platforms, while IncX-type plasmids (especially IncX3) remain important shuttles for *blaNDM* (53, 54). In our study, all *bla*NDM-positive *E. coli* isolates successfully transferred their carbapenemase gene to recipient strains by conjugation, with frequencies ranging from 10^−^⁴ per donor cell (53). These values are consistent with previously reported ranges for epidemic NDM-carrying plasmids, such as IncX3 backbones, which are well recognized for their capacity to mediate efficient horizontal gene transfer among Enterobacteriaceae.

However, because our analysis was based on short-read second-generation sequencing, which generates fragmented assemblies, we could not definitively determine the exact plasmid replicon(s) carrying *bla*NDM. This represents an important limitation and underscores the need for long-read or hybrid sequencing approaches to resolve complete plasmid structures in future studies. Taken together, our results place pediatric NDM-positive *E. coli* within this high-risk plasmid landscape: poly-replicon, IncF-enriched plasmids with accessory IncX, IncR, and IncHI elements create a genetic context favorable for capturing and mobilizing resistance cargo, thereby amplifying dissemination potential.

EspL is a type III secretion system effector and cysteine protease that cleaves RHIM-containing adaptor proteins (RIPK1/RIPK3, TRIF, and ZBP1), thereby inhibiting necroptosis and attenuating inflammatory signaling; *in vivo* evidence indicates that EspL promotes intestinal colonization (55). Given the small sample size and the multifactorial nature of biofilm formation, the observed association between *espL4* carriage and enhanced biofilm production should be regarded as preliminary and hypothesis generating only. Dedicated mechanistic studies are needed to determine whether *espL4* directly contributes to biofilm formation in NDM-producing *E. coli*.

In summary, our study provides an integrated genomic and phenotypic characterization of pediatric gut-derived NDM-positive *E. coli* isolates. These strains were dominated by *bla*NDM-5, carried extensive resistomes, and maintained a conserved set of virulence determinants that support colonization and persistence. The frequent presence of poly-replicon plasmids, particularly IncF-type backbones, underscores their high potential for resistance dissemination. Functionally, all isolates exhibited biofilm formation, siderophore activity, and epithelial cytotoxicity, with a hypothesis-generating signal linking *espL4* to enhanced biofilm production. Together, these findings confirm that the pediatric gut can harbor multidrug-resistant NDM-producing *E. coli* with colonization and emphasize the need for continued genomic surveillance and mechanistic studies to guide infection control and therapeutic strategies.

## Data Availability

The whole-genome sequencing data for the 25 NDM-producing *E. coli* isolates described in this study have been deposited in the National Center for Biotechnology Information (https://www.ncbi.nlm.nih.gov/sra/?term=) under the following accession numbers: SAMN54299078–SAMN54299099, SAMN54249838, SAMN54242561, and SAMN54242562.

## SUPPLEMENTAL MATERIAL

Supplemental material spectrum.03329-25-s0001.pdf Fig. S1 and S2.

ASM does not own the copyrights to Supplemental Material that may be linked to, or accessed through, an article. The authors have granted ASM a non-exclusive, world-wide license to publish the Supplemental Material files. Please contact the corresponding author directly for reuse.
