# Supplementary material for: Phenotypic and genomic characterization of NDM-producing Escherichia coli colonizing the pediatric gut in Shenzhen, China
Source: Microbiol Spectr. 2026 Feb 13;14(4):e03329-25. doi: 10.1128/spectrum.03329-25 (PMC13055255; doi:10.1128/spectrum.03329-25)
Supplement: Supplemental material — Fig. S1 and S2. [file spectrum.03329-25-s0001.pdf]

Fig S1

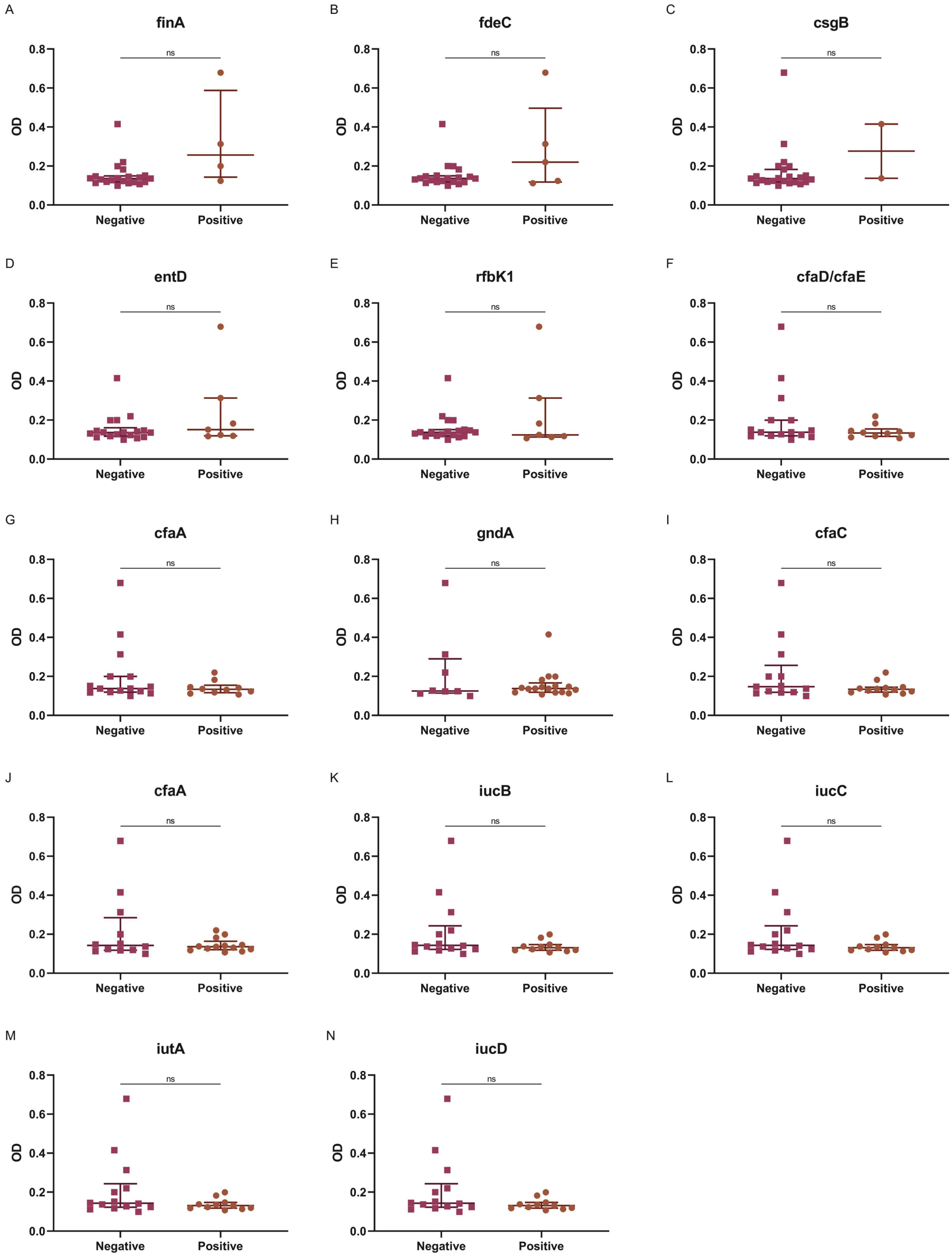

Fig S1. Scatter plots of OD595 values stratified by carriage of individual virulence genes. A finA, B fdeC, C csgB, D entD, E rfbK1, F cfaD/cfaE, G cfaA, H gndA, I cfaC, J cfaA, K iucB, L iucC, M iutA, N iucD. Each dot represents one isolate; the central line and whiskers depict the mean ± SD. Two-sided Mann–Whitney U tests were used and ns indicates not significant ($p \geq 0.05$).

Fig S2

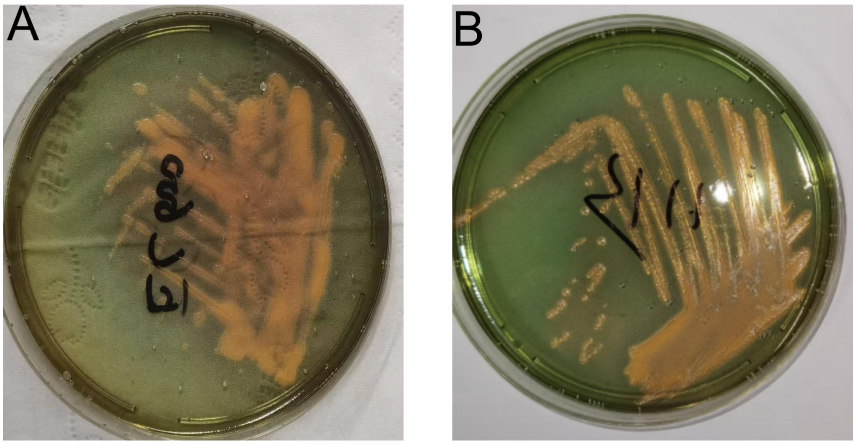

Fig S2. CAS assay of iron-chelating capacity . A shows the positive control EC600, and B shows the positive results of this assay.
